# Investigation of the clinical inter-observer bias in prostate fiducial marker image registration between CT and MR images

**DOI:** 10.1186/s13014-021-01865-8

**Published:** 2021-08-16

**Authors:** Emilia Persson, Sevgi Emin, Jonas Scherman, Christian Jamtheim Gustafsson, Patrik Brynolfsson, Sofie Ceberg, Adalsteinn Gunnlaugsson, Lars E. Olsson

**Affiliations:** 1 Radiation Physics, Department of Hematology, Oncology, and Radiation Physics , Skåne University Hospital, Klinikgatan 5, 221 85 Lund, Sweden; 2 Department of Translational Medicine, Medical Radiation Physics, Lund University, Carl Bertil Laurellsgata 9, 205 02 Malmö, Sweden; 3 Department of Medical Radiation Physics, Lund University, Barngatan 4, 222 85 Lund, Sweden

**Keywords:** Registration uncertainty, Inter-observer, Prostate cancer, Fiducial marker, Computed tomography, Magnetic resonance imaging, Image registration

## Abstract

**Background and purpose:**

Inter-modality image registration between computed tomography (CT) and magnetic resonance (MR) images is associated with systematic uncertainties and the magnitude of these uncertainties is not well documented. The purpose of this study was to investigate the potential uncertainty of gold fiducial marker (GFM) registration for localized prostate cancer and to estimate the inter-observer bias in a clinical setting.

**Methods:**

Four experienced observers registered CT and MR images for 42 prostate cancer patients. Manual GFM identification was followed by a landmark-based registration. The absolute difference between observers in GFM identification and the displacement of the clinical target volume (CTV) was investigated. The CTV center of mass (CoM) vector displacements, DICE-index and Hausdorff distances for the observer registrations were compared against a clinical baseline registration. The time allocated for the manual registrations was compared.

**Results:**

Absolute difference in GFM identification between observers ranged from 0.0 to 3.0 mm. The maximum CTV CoM displacement from the clinical baseline was 3.1 mm. Displacements larger than or equal to 1 mm, 2 mm and 3 mm were 46%, 18% and 4%, respectively. No statistically significant difference was detected between observers in terms of CTV displacement. Median DICE-index and Hausdorff distance for the CTV, with their respective ranges were 0.94 [0.70–1.00] and 2.5 mm [0.7–8.7].

**Conclusions:**

Registration of CT and MR images using GFMs for localized prostate cancer patients was subject to inter-observer bias on an individual patient level. A CTV displacement as large as 3 mm occurred for individual patients. These results show that GFM registration in a clinical setting is associated with uncertainties, which motivates the removal of inter-modality registrations in the radiotherapy workflow and a transition to an MRI-only workflow for localized prostate cancer.

## Background

A modern radiotherapy workflow for localized prostate cancer utilizes a combination of computed tomography (CT) images and magnetic resonance (MR) images for target definition. T2 weighted MR imaging (MRI) is currently preferred for prostate delineation due to the superior soft-tissue contrast [1]. The CT images, which provide electron density information, are traditionally used for dose calculation and patient set-up. To enable the combined use of CT and MR images in the workflow, image registration is needed. Mutual information (MI) [2–5] or landmarks such as gold fiducial markers (GFMs) [6, 7] are common approaches for image registration. The prostate volume, delineated in the MR images, is transferred to the CT images using the underlying image registration. Since the CT and MR images are acquired at different times, a change in patient anatomy and the internal organs can occur between images. A misalignment in the image registration will thus result in a systematic shift of the prostate volume used for treatment planning. This systematic uncertainty could severely impact the treatment if not accounted for in the planning target volume (PTV) margin, which renders image registration uncertainty important to investigate.

Since systematic uncertainties affect each treatment fraction [8] they are of highest importance to reduce. A potential reduction of image registration uncertainty, along with time and cost efficiency, have been motives for the clinical introduction of so-called MRI-only radiotherapy workflows. In these workflows, synthetic CT (sCT) images are generated from MR images and used throughout the workflow, eliminating the need for CT imaging [9, 10]. Image registration between different imaging modalities is therefore not needed. It has been reported that the systematic image registration uncertainty can be reduced by 1–2 mm [11]. Prostate cancer is a natural starting point for MRI-only treatments [12], and clinical implementations of MRI-only workflows have been presented [13–18]. The potential benefits of implementing an MRI-only workflow, i.e. reducing registration uncertainty, for localized prostate cancer has however been questioned, since a high image registration accuracy should be possible when GFMs are used for image registration of CT and MR images [19].

The magnitude of image registration uncertainty depends on how the CT and MR images are used in the workflow [19]. Different approaches for registration of MR and CT images of the prostate have been investigated by several groups, where GFM registration approaches in radiotherapy have shown results with average deviations below 2 mm [20, 21]. Huisman et al. showed that GFM surface-based registration yielded a higher precision compared to registration on the GFM center of mass (CoM), where 2 mm was considered the clinical goal [6]. MI and brachytherapy seed-based registration approaches have shown comparable results with an estimated uncertainty of 2 mm [3], a registration error of 2 mm [4], and overall accuracy of 1.5 mm [5]. Korsager et al. compared MI to manual landmark-based registration and found translational differences of the clinical target volume (CTV) ranging from 0 to 10 mm [2]. The motivation for MI has been higher work efficiency due to automatization [4] as well as lack of subjectivity in the method [3]. Landmark-based approaches rely on manual landmark selection, and are therefore argued to be inter-observer dependent [2]. GFMs together with CT-based imaging are however the preferred set-up strategy to account for inter-fractional prostate motion during the course of treatment [22] and are therefore commonly used.

Previous studies have been performed with relatively small study cohorts, comparing either two image registration methods or registrations to a ground truth image registration. How well these comparisons correlate to a clinical situation is not known. The resemblance with a clinical situation is important since the evaluation of an image registration method should resemble its potential clinical use. Factors such as stress and time constraints are common in daily clinical work, which may introduce a difference between a clinical situation and retrospective study results. So far, these factors have not been part of a study of GFM registration. The potential differences between the study set-up and the current clinical situation should be considered during analysis. Current literature is also lacking studies of the inter-observer variability when GFM registration approaches are used in a clinical setting. The use of a ground truth image registration, constructed to evaluate the uncertainty in a method, merely sets two approaches in contrast to each other. Instead of comparing different methods, the clinical image registration uncertainty would be more appropriately represented by the user bias within the chosen method and set this in perspective to the clinical use of the method.

The aim of this study was to investigate the registration uncertainty and estimate the inter-observer image registration bias in standard GFM registration approach currently in clinical use at Skåne University Hospital, Sweden, for localized prostate cancer patients.

## Methods

### Patients and image acquisition

Forty-two patients, previously treated for prostate cancer at the radiotherapy department at Skåne University Hospital, were enrolled in the study. Median age and weight were 72 years [59 to 82 years] and 88.5 kg [64 to 117 kg], respectively. All patients were prescribed radiotherapy to the prostate gland, with or without involvement of the vesicles or iliac lymph nodes, up to 78 Gy in 39 fractions. Three cylindrically shaped in-house manufactured GFMs (1 mm in diameter and 5 mm long) were inserted into the prostate using a biopsy needle approximately two weeks prior to CT and MR imaging. These GFMs were used for image registration between the CT and MR images prior target delineation and for patient set-up verification before treatment delivery.

A flat tabletop with patient immobilization of feet and knees was used during CT and MR imaging. CT imaging was performed using a Siemens Somatom Definition AS+ (Siemens Healthineers, Erlangen, Germany) with a 3 mm slice thickness. MR imaging was performed using a GE Discovery 750w 3.0T (GE Healthcare, Chicago, Illinois, USA) directly after the CT imaging. A 16-channel GEM Anterior Array coil, positioned on stiff coil bridges, was used for MR imaging. A multi-echo gradient echo (MEGRE) sequence, previously described [23], was acquired for fiducial marker identification and subsequent image registration with a 2.8 mm slice thickness. T2 weighted MR images (transversal, sagittal and coronal projections) were acquired for target delineation.

### Image registration

**Fig. 1:**
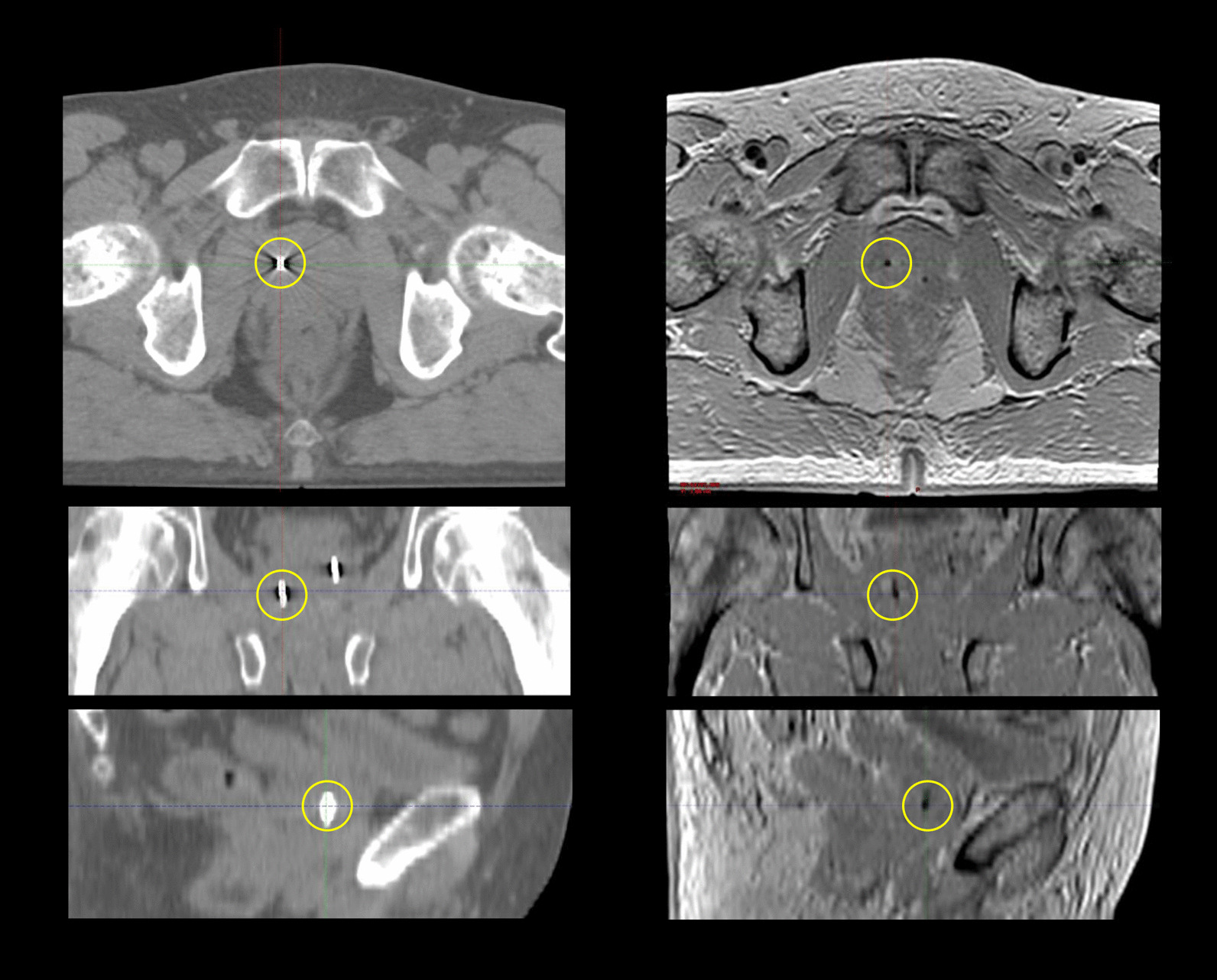
CT images (left) and multi echo gradient echo (MEGRE) MR images (right) used for gold fiducial marker (GFM) registration. The GFM, highlighted in yellow circles, generate a streak artefact in the CT images and a signal void in the MR images. Images are shown in transversal (top row), coronal (middle row, calculated projection) and sagittal (bottom row, calculated projection) planes

Image registration was performed using point match registration in Eclipse™ treatment planning system (TPS) (Version 13.6 or 15.6, Varian Medical systems, Palo Alto, CA, USA). The point match registration is a landmark-based registration method, which performs a translation and rotation of a target image to a source image in six degrees of freedom (x, y, z, pitch, rotation and roll). In this study, the MEGRE MR image was used as target image, and the CT image was used as source image. The CoM of three GFMs, identified in each image, were used as reference for the image registration. The point match registration outputs a parameter matrix, which is used to transform the MR image to the CT image geometry. According to the predefined clinical guideline at Skåne University Hospital, the image registration was initiated by identification of the CoM of three GFMs in the CT and MR images respectively (Fig. 1). CT and MR images were simultaneously visible on the observer screen setup during identification. The GFM CoM was restricted to be defined in a physical slice of the CT and MR images, and not in between slices. If the GFM CoM was found to be positioned between two slices in the images, the observers were instructed to choose what they considered to be the most centrally positioned physical slice. The manual identification was followed by an automatic registration process. The alignment of the CT and MR images was visually reviewed using split window, moving window or image blend after execution of the image registration. If the alignment was found unacceptable by the observer’s visual assessment or if the point match quality, given by the TPS, exceeded 1.5 mm, the automatic image registration was repeated after manually changing the position of one or more of the GFMs CoM.

#### Clinical baseline

Registrations between CT and MR images routinely created and used for target delineation in the clinical workflow, are in this study referred to as the clinical baseline. The clinical baseline registrations were performed by the four MR technicians, hereinafter called observers, between the 1st of October 2018 and 30th of April 2019. The four observers were all experienced in image registration and performed registration of CT and MR images daily according to a clinical guideline. Observers 1, 2, 3 and 4 performed 11, 16, 8 and 7 registrations respectively, in the clinical baseline. The observers had no knowledge of the upcoming study at the time of the clinical baseline registrations. Following the registration, the CTV was defined by an oncologist, using a blended view of the registered CT images and T2 weighted MR images. The clinical baseline registrations were thereby independently validated by an oncologist in connection to the target delineation process. Time stamps for each image registration were recorded in the clinical database.

#### Observer registrations

In October 2019, the four observers individually repeated all image registrations for the whole study population. This resulted in a total of 504 identified GFMs and 168 new image registrations, further referred to as the observer registrations. All observer registrations were performed in a single day according to the clinical guideline. At the day of the study, prior to the observer registrations, an introduction to the method and aim of the study was given to the observers. To verify given instructions, three test patient cases were provided to the observers and registrations were performed according to given instructions. The test cases contained data similar to the study population, and the observers were given the opportunity to ask questions regarding the instructions to a study coordinator (EP). The observers were instructed to perform the registrations consecutively, starting from patient 1 to patient 42, and approve the image registration in the TPS after each patient. The observers could not see each other’s registrations or the clinical baseline registrations during the study. The approvals created and recorded start and finish time stamps for the observer registrations for each patient. This enabled investigation of whether the study resembled a clinical situation in terms of time spent on performing the registrations.

### Analysis

The observer GFM identification bias in CT and MR images was investigated by comparing the spatial coordinates of the GFMs identified by the four observers in the CT and MR images. The spatial coordinates of the three GFMs, identified in the CT and MR geometry respectively, were saved as a DICOM radiotherapy structure set for each patient and observer during the manual identification. The absolute difference in identified GFM CoM between the observers was investigated for anterior-posterior (AP), left-right (LR) and cranial-caudal (CC) directions. The absolute difference between two observers was calculated by subtracting the spatial coordinates of each identified GFM CoM between the two observers. This resulted in a total of 6 absolute differences for each GFM CoM and each anatomical direction.

**Fig. 2:**
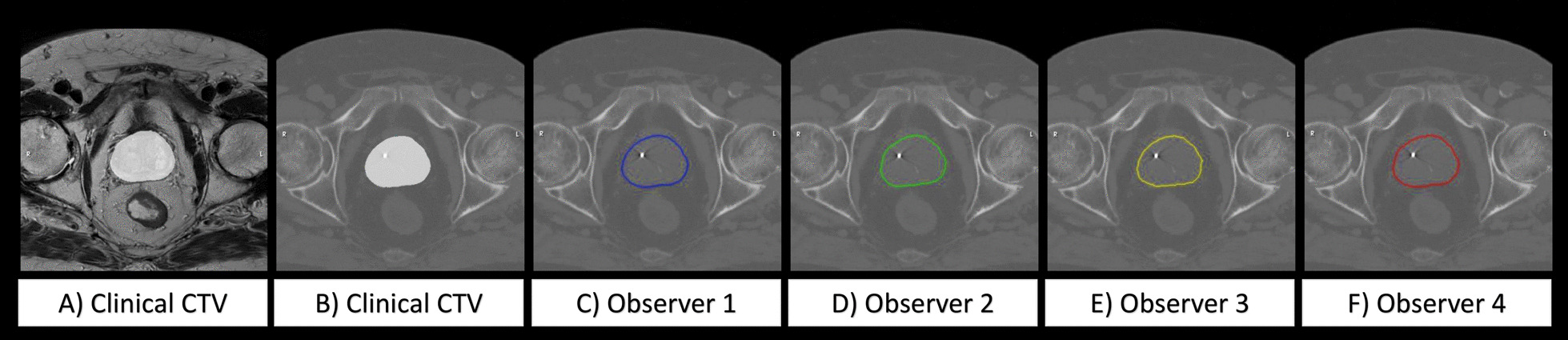
An example of a patient where the clinical target volume (CTV) has been transferred from the MR geometry (**A**) to the CT geometry (**B**–**F**). All images are shown in the corresponding transversal slice through the center of the prostate volume. The clinical CTV is illustrated as a white structure in the center on the MR image (**A**) and on the CT image (**B**). The corresponding CTV is shown for observer 1, 2, 3 and 4 in blue, green, yellow and red contours (**C**–**F**)

The uncertainty in an image registration method can only be evaluated when all parameters of the image registration matrix are combined. Therefore, the CTV was transferred from the MR to the CT geometry using the four observer registrations for each patient. The CT and MR images were first resampled to a common voxel size of 0.469 × 0.469 × 1 mm in AP, LR and CC directions. Secondly, the CTV, including either the prostate gland alone or combined with the vesicles, was transferred from the MR images to the CT images using the observer and clinical baseline registrations, respectively, for each patient. This created one clinical CTV position and four observer CTV positions in the CT-geometry (Fig. 2) for each patient.

The distance vectors between the CoM of the clinical CTV to the CoM of each observer CTVs were calculated for each patient, which will be referred to as the CTV CoM vector displacement. The DICE-indices and Hausdorff distances for each observer CTV compared to the clinical CTV were calculated. Analysis was performed using MICE Toolkit (Version 1.1.1, NONPI Medical, Umeå, Sweden).

Paired samples Wilcoxon test was used to investigate if there was a statistically significant difference between the GFM CoM identification in CT and MR images in the three anatomical directions. The paired samples Wilcoxon test was also used to examine if there was a statistically significant difference between any of the observers’ CTV CoM vector displacements from the clinical baseline, and to investigate if there was a statistical difference between the time spend on the registrations. All tests were performed in R with a significance level of *p* < 0.05 (Version. 3.5.1, RStudio, Boston, MA, USA).

## Results

**Fig. 3:**
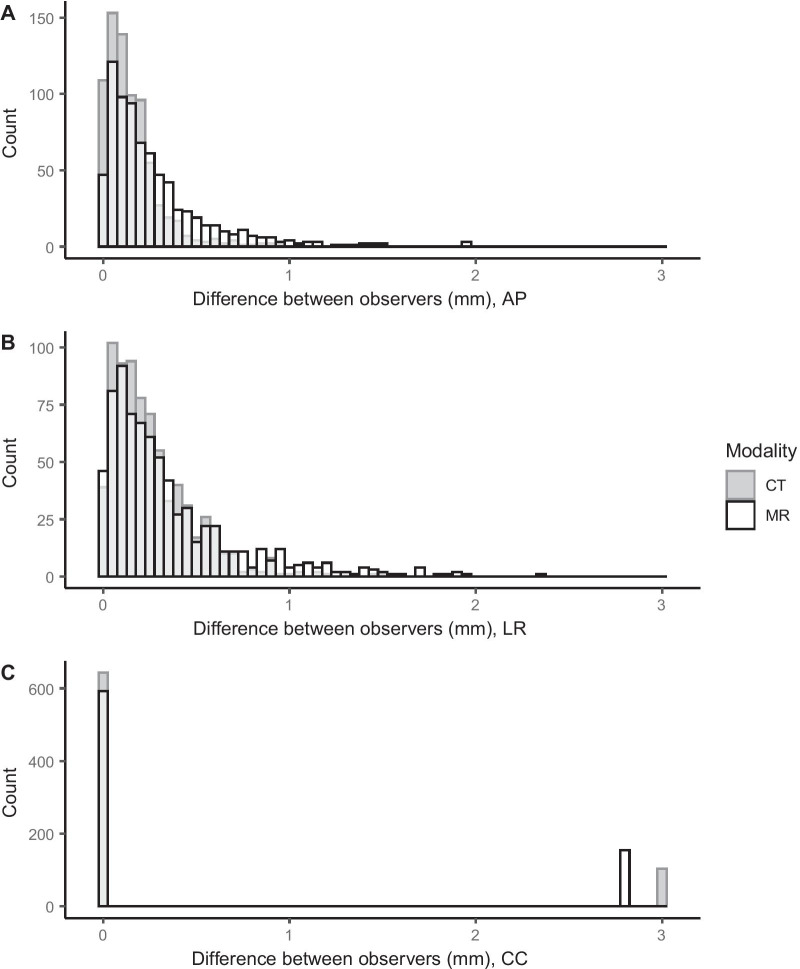
Absolute difference for each identified gold fiducial marker (GFM) center of mass (CoM) between observers, displayed in anterior-posterior (**A**), left-right (**B**) and cranio-caudal (**C**) directions. On the x-axis, the absolute difference between observers in mm is shown for identification in CT images (dark gray) and MR images (white with black outline). On the y-axis the number of observations in each bin are shown. Bin width was 0.05 mm for all graphs. Bins with an overlap of the CT and MR are colored in light gray

Observer 2 completed one image registration incorrectly, which was noted during analysis. This led to a large deviation of − 73.0 mm in the CC direction. This observer registration was excluded. The median observer difference in identified GFM CoM in AP, LR and CC, given as absolute difference in mm [range], was 0.1 [0.0–0.9], 0.2 [0.0–1.5] and 0.0 [0.0–3.0] in CT images, and 0.2 [0.0–2.0], 0.2 [0.0–2.3] and 0.0 [0.0–2.8] in MR-images (Fig. 3). There was a statistically significant difference between identification in CT and MR images in the AP and LR direction (*p* < 0.001) while no statistically significant difference was found in the CC direction (*p* = 0.24).

**Fig. 4:**
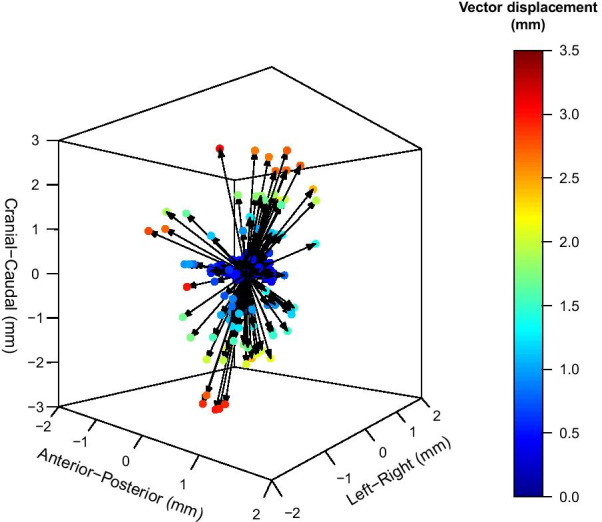
Clinical target volume (CTV) center of mass (CoM) vector displacement from the clinical baseline. The CTV CoM vector displacements for 167 observer registrations are displayed as colored dots ranging from dark blue (0 mm vector displacement) to dark red (3.5 mm vector displacement)

The median CTV CoM vector displacement from the clinical baseline for all observers was 0.7 mm [range: 0.0–3.1] (Fig. 4). The corresponding median for observers 1 to 4 individually was 0.8 mm [range: 0.1–3.0], 0.6 mm [range: 0.0–3.0], 1.0 mm [range: 0.0–3.0] and 0.7 mm [range: 0.1–3.1]. There was no statistically significant difference between the observer CTV CoM vector displacements (*p* > 0.65). Observer registrations yielding a CTV CoM vector displacement larger than or equal to 1.0 mm, 2.0 mm and 3.0 mm were 46% (76/167), 18% (30/167) and 4% (6/167) respectively (Fig. 4). Vector values were rounded to one decimal place prior to calculation. The six deviations larger than or equal to 3.0 mm were found for 4 different patients, number 12, 13, 26 and 36. Two of the patients had deviations for two observers. This meant that the maximum deviation was not strictly related to a single patient. Median DICE-index for the observers was 0.94 [range: 0.70–1.00] and median Hausdorff distance for the observers was 2.5 mm [range: 0.7–8.7].

**Fig. 5:**
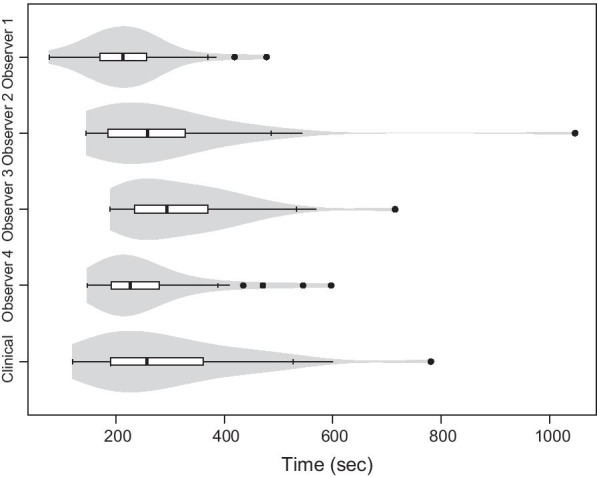
Time required for the image registrations. The times for the observer registrations are shown in order 1 to 4 from the top and at the bottom row the clinical baseline registration time is shown. The figure includes a boxplot overlaid on a violin plot. The medians are defined as solid black lines in the boxes and outliers as solid black dots. The width of the violin plot indicates the frequency

**Fig. 6:**
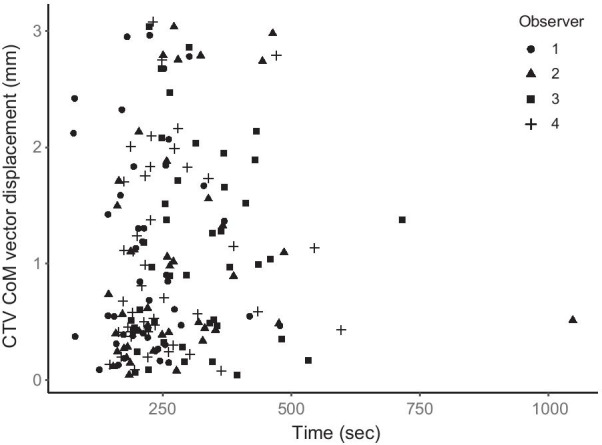
The relation between registration time and the clinical target volume (CTV) center of mass (CoM) vector displacement for observer 1 (circles), observer 2 (triangles), observer 3 (squares) and observer 4 (plus signs). Time is displayed in seconds on the x-axis and the CTV CoM vector displacement in mm on the y-axis. All observers were represented in the range of displacements and times. Observer 1 was clearly in the lower range of registration times, also seen in Fig. 5, but was not exclusively part of the lower nor higher range of CTV displacements

The median registration time for the clinical baseline registrations, given in min:s [range], was 4:17 [2:00–13:01]. The median times for each of the observer registrations, given in min:s [range] were 3:33 [1:17–7:58], 4:18 [2:25–17:27], 4:55 [3:09–11:55] and 3:46 [2:27–9:57] (Fig. 5). Two of the clinical baseline registrations and one observer registrations had unreliable time stamps and were excluded. A statistically significant difference was detected between observer 1 and 2 (*p* = 0.03), 1 and 3 (*p* < 0.001) and 1 and 4 (*p* = 0.03). Observer 1 were in general faster than the other observers. A statistically significant difference was also seen between observers 3 and 4 (*p* = 0.01). Observer 1 had a statistically significant difference from the clinical registration time (p = 0.01) while no statistically significant difference was detected for observers 2–4 (*p* = 0.48, *p* = 0.17 and *p* = 0.16). The CTV CoM vector displacement as a function of time is presented in Fig. 6.

## Discussion

In this paper, a CT and MR image registration approach for localized prostate cancer using GFMs was investigated. Image registrations for 42 prostate cancer patients were carried out by four experienced observers according to clinical practice. The observer bias in GFM identification in CT and MR images was investigated and observer registrations were compared to a clinical baseline. The uncertainty was investigated by converting the image registrations into a CTV displacement from the clinical baseline. To our knowledge, this is the first study to present the propagation of GFM identification observer bias into image registration differences for multiple observers compared to a clinical baseline.

The results showed that the investigated GFM image registration approach was observer-dependent for individual patients. The CTV CoM was displaced up to 3.1 mm and the Hausdorff distance ranged up to 8.7 mm. These deviations are in the same order of magnitude as CTV to PTV margins currently in use for prostate cancer radiotherapy [24]. One observer made an accidental mistake in one image registration, leading to a large difference compared to the clinical baseline and the other observers. Although this was an obvious mistake, it was not noted by the observer. This event shows that major operator mistakes can occur, which highlights the need for quality assurance (QA) programs and independent checks of the performed image registration in clinical routine, as previously recommended by the AAPM task group no. 132 [25]. The current findings further motivate the clinical introduction of MRI-only workflows for localized prostate cancer, which eliminate the need for inter-modality image registrations and associated uncertainties. MRI-only radiotherapy may allow for reduced CTV to PTV margins in the future and thereby decrease the dose to normal tissue and reduce toxicity.

The observer differences in identified GFM CoM were overall larger in MR images than in CT images and there was a statistically significant difference in AP and LR-directions. Median observer differences were below 0.2 mm in all directions. The largest deviation, which was found in the CC-direction, showed no statistical difference between CT and MR images. In the present study, very experienced observers were involved, and similar MR and CT slice thicknesses were used. This could potentially have contributed to the small differences seen for GFM identification in the two modalities. This demonstrates the value of training and education in the use of MR images in radiotherapy and particularly for GFM identification.

The vector displacement from the clinical CTV to the observer CTV varied from 0.0 to 3.1 mm and a variation was seen in DICE-indexes as well Hausdorff distances. The median CTV CoM vector displacement of 0.7 mm found in the present study is slightly lower than the range of previously reported image registration uncertainties of 1–3 mm [2–5, 20, 21]. These studies did however compare different methods and did not investigate the clinical use of the methods, as in the present study. A CTV CoM vector displacement of 2 mm or more was found in 18% of the observer registrations. This means that in about one out of five image registrations based on GFMs, there is a risk of introducing a CTV CoM vector displacement of 2 mm or above as a result of observer bias. These findings show that a well-established image registration method operated by experienced staff, as in the present study, can still introduce large differences for individual patients. Although no statistical difference was found between the observers CTV displacement, this does not exclude the risk that individual registrations can result in large deviations.

A statistically significant difference in registration time was detected between observer 1 and the remaining observers as well as the clinical registration. In Fig. 5 its clear that observer 1 was faster than the other observers. Observer 2–4 did not differ statistically significant from the clinical registration time. The fast registration time by observer 1 could be a result of the study setup, where a stress and competing factor could have affected the results. Nevertheless, the registrations by observer 1 did not differ statistically significant from the remaining observers in terms of CTV displacement, also seen in Fig. 6. One could expect a study to result in statistically significant longer image registration times compared to a clinical situation, due to factors such as stress and time constraints in a clinical situation, although this was not the case in the present study. The observer registrations in this study can therefore be considered to resemble a clinical situation in terms of time. All retrospective image registrations in the present study were carried out in a single day. Unnecessarily long time was therefore not spent on each patient, which might be the case if the study spans over several days. The observers were not permitted to redo any of the image registrations after approval, which also mimics a clinical situation. However, the number of registrations performed during the one-day study time, exceeded the number of registrations normally completed during a working day. The results showed no indication of bias introduced by the long study day, with maximum deviations present both early and later during the day.

Huisman et al. included inter-observer dependency in their investigation of two GFM registration approaches and estimated a 2 mm uncertainty in both methods [6]. In contrast to our study, their observers had no previous image registration experience, which could potentially result in a higher deviation. Further, all image registrations were performed in a study set-up and they did not compare their results to a clinical baseline. Korsager et al. discussed the problem of a non-existing ground truth in comparisons of registration approaches. They suggested the use of a clinical image registration to eliminate observer bias in favor of using a created ground truth [2]. This is in accordance with the method used in the present study, where the clinical image registrations serve as a baseline. However, this baseline should not be misinterpreted as a ground truth but should rather be a tool to investigate observer bias in a clinical situation.

This study has several limitations. The observer registrations in the present study were carried out with observers being informed about the study. This can be considered a limitation since this does not mimic a true clinical situation. A representation of a true clinical bias could be achieved by performing unnoticed dummy-runs in a clinical setting. The slice thickness and the restriction of GFM identification in a physical slice are also limiting factors, and discrepancies in the order of the slice thickness (3.0 mm in CT images and 2.8 mm in MR images) are expected. Nevertheless, slice thickness is a limiting factor present in everyday clinical work, and a smaller slice thickness could potentially reduce the uncertainty. The GFM identification observer bias presented in this study is a variation to be expected in the dual-modality workflow when GFMs are used for inter-modality image registration. The Hausdorff distance analysis is also limited by the image resolution, as the image resolution limits the smallest detectable difference in this analysis. It is worth noting that the six parameters in a registration matrix all depend on each other. The result of an image registration is thereby first seen when the effects from all parameters are combined. Differences in individual image registration parameters of the matrix can thus look extreme when comparing two observers, and the analysis should be complemented by combining all six parameters. The clinical impact of image registration observer bias is preferably represented by the displacement of the CTV, since this combines all six matrix parameters into one clinically relevant measure, as demonstrated in the present paper.

## Conclusions

The results of this study showed that GFM registration of CT and MR images in a clinical setting was associated with uncertainties. Median CTV CoM displacement was 0.7 mm and displacements as large as 3.0 mm occurred for individual patients due to observer bias in the GFM identification process and the finite thickness of the slice. This uncertainty should be considered in PTV margin calculation. The results of this study emphasize the potential gain of removing inter-modality image registrations and motivates the implementation of an MRI-only workflows for localized prostate cancer patients.

## Data Availability

The datasets generated during/or analyzed during the current study are not publicly available due to patient privacy concerns and institutional regulations.

## Abbreviations

AP: Anterior–posterior
CC: Cranio-caudal
CoM: Center of mass
CT: Computed tomography
CTV: Clinical target volume
GFM: Gold fiducial marker
LR: Left-right
MEGRE: Multi echo gradient echo
MI: Mutual information
MR: Magnetic resonance
MRI: Magnetic resonance imaging
PTV: Planning target volume
sCT: Synthetic CT
